# Becker’s nevus

**DOI:** 10.11604/pamj.2022.41.142.33417

**Published:** 2022-02-17

**Authors:** Krishna Prasanth Baalann, Mahalakshmi Krishnan

**Affiliations:** 1 Department of Community Medicine, Sree Balaji Medical College and Hospital, Bharath Institute of Higher Education and Research, Chennai, Tamil Nadu, India,; 2 Department of Microbiology, Sree Balaji Dental College and Hospital, Bharath Institute of Higher Education and Research, Chennai, Tamil Nadu, India

**Keywords:** Becker’s nevus, hyperpigmentation, hypertrichosis

## Image in medicine

Becker's nevus is also known as Becker melanosis. It is a benign lesion which can be presented as congenital or acquired with hairless or hypertrichotic lesions. It's a rare case which affects mainly male individuals. It is often pigmented and gets darker by time and excessive hair growth can be seen over it. A 29-year-old transgender patient presented with hyperpigmentation with the lesion which started at the age of 15 as a small hyperpigmented macule. The lesion increased gradually to form giant patches. On examination a right-side hyperpigmentation involving the anterior chest, shoulder, scapular region, upper arm with hypertrichosis and irregular margins. On histopathological examination, hyperpigmentation of the basal layer and melanophages were present in the upper dermis. A diagnosis of Becker’s nevus was made based on the clinical appearance and histopathological findings. After counseling the patient about the disease, laser therapy was referred and regular follow-up was advised.

**Figure 1:**
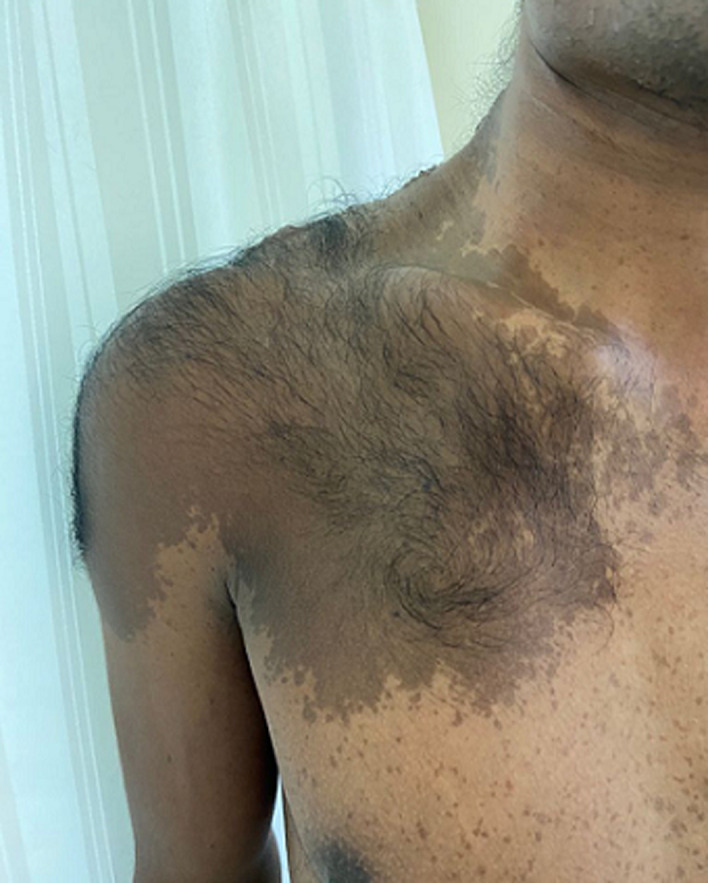
right side hyperpigmentation involving the anterior chest, shoulder, scapular region

